# Key Fibrogenic Signaling

**DOI:** 10.1007/s40139-015-0077-z

**Published:** 2015-04-05

**Authors:** Weichun He, Chunsun Dai

**Affiliations:** Center for Kidney Disease, Second Affiliated Hospital, Nanjing Medical University, 262 North Zhongshan Road, Nanjing, 210003 China

**Keywords:** Fibrosis, Smad, Mitogen-activated protein kinase, Phosphoinositide 3 kinase, Wnt/β-catenin, Sonic hedgehog

## Abstract

Fibrosis is defined as an excessive accumulation of extracellular matrix components that lead to the destruction of organ architecture and impairment of organ function. Moreover, fibrosis is an intricate process attributable to a variety of interlaced fibrogenic signals and intrinsic mechanisms of activation of myofibroblasts. Being the dominant matrix-producing cells in organ fibrosis, myofibroblasts may be differentiated from various types of precursor cells. Identification of the signal pathways that play a key role in the pathogenesis of fibrotic diseases may suggest potential therapeutic targets. Here, we emphasize several intracellular signaling pathways that control the activation of myofibroblasts and matrix production.

## Introduction

Fibrosis, characterized by excessive accumulation of extracellular matrix (ECM) in and around inflamed or damaged tissue, leads to tissue destruction,permanent scarring, and organ malfunction [1••]. Although fibrotic conditions may result from diverse causes, it is generally thought that an initial injury activates a repair process that aims to restore the original tissue structure, and that a failure to delicately regulate this process results in sustained fibroblast activation, matrix deposition, and tissue devastation [2]. Fibrosis is part of progressively chronic diseases in parenchymal organs throughout the body, and the fibrotic process plays an essential role in the deterioration of these organs [1••, 3••, 4]. Few effective therapies to halt tissue fibrosis, or to reverse it, are available in clinical practice [5••]. Consequently, it is important to thoroughly understand the cellular and molecular mechanisms of fibrogenesis, not only to acquire novel insights into the pathogenesis of the fibrotic process, but also to further exploit efficient strategies to treat patients with fibrotic disorders.

Fibrogenesis is a dynamic and progressive process in which nonresolving inflammation following a persistent injury sets the stage for fibrosis and triggers the activation of matrix-producing myofibroblasts differentiated from a variety of precursor cell types through different mechanisms, including activation of resident fibroblasts and pericytes, phenotypic transition of epithelial and endothelial cells, phenotypic modulation of vascular smooth muscle cells, and recruitment of circulating multipotent monocytes and fibrocytes [5••]. The mechanisms governing the fibrotic process have been studied in great detail in the past years. Regardless of the initiating event, a feature common to all fibrotic diseases is the activation of myofibroblasts, key mediators in fibrotic tissue remodeling. Once activated, myofibroblasts produce and secrete components of ECM, such as collagen and fibronectin [5••, 6•, 7•]. Because fibrogenesis may be evoked by a variety of stimuli, it is conceivable that a diverse array of signaling networks and mediators might be involved in regulating the progression of fibrosis. The scope of this review is limited to several key intracellular signal transduction pathways that are essential in controlling a host of transcription regulators and signaling mediators that are necessary for fibrogenesis. It is hoped that a precise delineation of these fibrotic signaling cascades will yield further insights into the pathogenesis of fibrosis and lead to the identification of novel targets for therapy.

## Smad Pathway: Canonical Pathway of Transforming Growth Factor-β Signaling

Transforming growth factor-β (TGF-β) is a profibrotic factor and a central mediator of fibrogenesis. The classical Smad-dependent pathway for TGF-β signaling occurs when TGF-β receptor type 2, which is constitutively active, transphosphorylates and forms a complex with the TGF-β-bound TGF-β receptor type 1. This complex then phosphorylates serine residues of cytoplasmic receptor-activated Smad (R-Smad), a complex of Smad2 and Smad3. These two heterodimerize and bind to the common mediator Smad (Co-Smad) Smad4, and the whole complex translocates across the nuclear membrane to interact with specific *cis*-acting elements in the regulatory regions of its target genes [8••], recruiting coactivators such as p300 and CBP; corepressors such as c-Ski, SnoN, transforming growth-inhibiting factor, and Smad nuclear-interacting protein 1; or transcription factors such as AP-1 and Sp1 to modulate gene expression [4]. Inhibitory Smad (I-Smad) Smad6 or Smad7, acting as negative regulators, not only antagonizes the TGF-β/Smad pathway by binding to TGF-β1 or competing with activated R-Smad for binding to Co-Smad, but also recruits the E3 ubiquitin–protein ligases Smurf1 and Smurf2, which target Smad proteins for proteasomal degradation, thereby blocking Smad2/3 activation, facilitating receptor degradation, and eventually terminating Smad-mediated signaling [4, 8••, 9–11].

A large amount of evidence shows that Smad signaling plays a crucial role in governing TGF-β-induced fibrosis. A variety of genes for mediating myofibroblast activation and epithelial–mesenchymal transition (EMT) are the downstream targets of TGF-β/Smad signaling, including connective tissue growth factor (CTGF), Snail, α-smooth muscle actin (α-SMA), collagen IA2, inhibitor of differentiation 1 (Id1), Wnt, matrix metalloproteinase 2 (MMP-2), integrin-linked kinase (ILK), β1-integrin, and PINCH-1 [12–15, 16•]. Mounting studies indicate that the canonical Smad2/3 pathway induces transition of the increased number of fibroblasts into active myofibroblasts and EMT, and acts on the latter cells as transcriptional activators of multiple ECM proteins as well as regulators of matrix accumulation [17].

The necessity of Smad signaling in fibrogenesis is illustrated clearly in Smad3 knockout mice [4, 16•]. Mice lacking Smad3 are protected from bleomycin-induced skin and lung fibrosis, dimethylnitrosamine-induced liver fibrosis, renal interstitial fibrosis due to unilateral ureteral obstruction, skin fibrosis following irradiation, and cardiac fibrosis [18–23]. In addition, Smad4-deficient mice also are protected from renal fibrosis after obstructive injury [8••, 24]. Conversely, I-Smad, such as Smad7, counteracts TGF-β-induced fibrosis. Overexpression of Smad7 blocks bleomycin-induced lung fibrosis and bile duct ligation-induced liver fibrosis [25, 26]. Dysregulation of SnoN, Ski, or Smurf2 may give rise to deviant TGF-β/Smad signaling in the pathogenesis of kidney fibrosis [27, 28].

## Mitogen-Activated Protein Kinase Pathway: Erk, p38, and Jnk

Besides activating the Smad-dependent pathway, TGF-β also can signal in a noncanonical manner. One of the Smad-independent pathways is the mitogen-activated protein kinase (MAPK) family. As the ubiquitous intracellular serine/threonine kinases, MAPK family members can transmit extracellular signals from cell surface receptors to intracellular targets, ultimately activating or inhibiting nuclear transcription factors and determining the cell’s fate [4]. MAPKs contain three major subfamilies: the extracellular signal-regulated kinases (Erk1 and Erk2), the p38/MAP kinases (α, β, γ, and δ), and the stress-activated protein kinases known as c-Jun N-terminal kinases (Jnk1, Jnk2, and Jnk3). All three MAPK pathways may be activated by TGF-β, and signaling through these cascades can further regulate the expression of Smad proteins and mediate Smad-independent TGF-β responses [9]. These three MAPK pathways are all involved in TGF-β-induced fibrosis [8••, 9, 29, 30].

TGF-β induces phosphorylation on TGF-β receptors 1 and 2 and/or Shc, which recruit Grb2/Sos to activate Erk through membrane-anchored Ras and downstream MAPK cascades [8••, 31]. Depending on the cell type studied, the activation of Erk can up- or down-regulate Smad signaling activity, whereas Jnk or p38 generally promote TGF-β-stimulated responses [4]. The TGF-β-Erk axis can mediate the transcription of genes controlling the EMT process, CTGF expression, and collagen I production by working with the Smad-dependent pathway, whereas Erk also can repress R-Smad activity through phosphorylation [4, 8••, 32, 33]. Together with Smads, the activated JnK/p38 pathways regulate TGF-β-induced fibroblast differentiation into myofibroblasts [8••, 30].

## Phosphoinositide 3-Kinase Pathway: Akt/mTOR and PAK2/c-Abl

The phosphatidylinositol 3-kinase (PI3K) pathway is another non-Smad pathway contributing to TGF-β-induced fibrosis. It induces two profibrotic pathways: Akt–mammalian target of rapamycin (mTOR) and p21-activated kinase 2 (PAK2)/Abelson kinase (c-Abl) [4, 8••, 9, 34].

Akt is a serine/threonine kinase that can engage multiple downstream signaling substrates and pathways. One of its downstream targets is Tuberin [tuberous sclerosis complex 2 (TSC2)], which binds to hamartin (TSC1). TSC1 contributes stability and prolongs the half-life of TSC2 within this complex, and TSC2 becomes phosphorylated and thereby inactivated. TSC2 functions as a GTPase-activating protein (GAP) specifically for Ras homolog enriched in brain (Rheb) [17, 35]. Rheb exists in either an active or inactive GDP-bound state, and Rheb–GTP activates target of rapamycin complex 1 (TORC1) [36–38]. TORC1 consists of several protein components, including mTOR itself, the regulatory-associated protein of mTOR (Raptor), and mLST [39, 40]. Active (nonphosphorylated) TSC2 converts mTORC1-activating Rheb–GTP to Rheb–GDP. Thus, active TSC2 is an inhibitor of mTORC1, and loss of TSC2 activity by phosphorylation increases mTORC1 activity. This activates downstream substrates, including p70 S6 kinase (S6K), a translational activator of many proteins, including cell cycle proteins, and hence, proliferation [17]. The Akt–mTORC1–P70S6K branch pathway contributes to fibroblast proliferation and myofibroblast differentiation. Abnormal activation of mTORC1 is involved in the pathogenesis of fibrotic disorders. Activation of mTORC1 induced by TGF-β is essential for its profibrotic effects on collagen production, whereas pharmacologic and genetic manipulation that decreases mTORC1 activity prevents fibrotic changes [17, 26, 41]. Rheb/mTORC1 signaling may promote activation of kidney fibroblasts and contributes to the development of interstitial fibrosis [38].

PI3K also acts as a branch point in response to TGF-β, leading to activation of PAK2/c-Abl. The latter not only stimulates collagen gene expression in normal fibroblasts, but also induces fibroblast proliferation, thereby increasing the number of myofibroblast precursors [42]. PAK2/c-Abl promotes fibrosis through its downstream mediators, including PKCδ/Fli-1 and early growth response (Egr)-1, -2, and -3 [8••, 43•, 44]. Unrestrained TGF-β activity might be associated with aberrantly sustained c-Abl activation, leading to Erk1/2-dependent upregulation and persistent expression of Egr-1 in fibroblasts [45]. Sustained Egr-1 expression in target tissues in turn would induce or perpetuate fibrotic responses. Activation of the c-Abl–Egr-1 pathway, presumably through PI3K and PAK2, represents an important novel mechanism for mediating TGF-β responses in fibroblasts [45].

## Rho GTPase Signaling Pathway

Rho GTPases, a subfamily of small GTP-binding proteins belonging to the Ras superfamily, modulate the actin cytoskeleton. Their activity is regulated by Rho guanine nucleotide exchange factors. The latter can interact directly with Rho proteins, allowing exchange of GDP for GTP [8••]. Rho is activated due to GTP-bound state and it can interact with downstream effector proteins, most notably Rho-associated, coiled-coil containing protein kinase (ROCK) and mouse diaphanous-related formin 1 (mDia1), which together initiate and stabilize actin stress fibers. Mechanistically, mDia1 induces F-actin filament nucleation, whereas phosphorylated ROCK regulates stabilization of F-actin through multiple downstream target genes [8••]. Hence, the amount of F-actin is increased, resulting in a decrease in G-actin monomer-free myocardin-related transcription factor (MRTF) to travel into the nucleus, where it cooperates with serum response factor (SRF) to induce gene transcription [8••].

Multiple target genes for MRTF/SRF are known drivers of fibrosis, including CTGF, α-SMA, and collagen [46, 47]. SRF-mediated gene transcription is crucial for the induction and maintenance of myofibroblast differentiation [48–50]. Of interest, Rho GTPase signaling with its downstream gene transcription mechanism MRTF/SRF seems to play a convergent role in pathways downstream of TGF-β, lysophosphatidic acid (LPA), endothelin 1 (ET-1), and integrins in fibrosis [8••]. It suggests that the Rho/MRTF/SRF transcriptional pathway may be a therapeutic target for fibrotic diseases [8••].

## Canonical Wnt/β-Catenin Signaling

Wnt proteins deliver their signal across the plasma membrane by interacting with Fizzled receptors and coreceptors, members of the LDL receptor-related protein 5/6. Once Wnts bind to their receptors/coreceptors, they initiate a chain of downstream signaling events implicating Disheveled, axin, adenomatosis polyposis coli (APC), casein kinase 1 (CK-1), and glycogen synthase kinase 3β (GSK-3β), leading to dephosphorylation of β-catenin [16•]. Escaping from degradation mediated by the ubiquitin/proteasome system stabilized β-catenin accumulates in the cytoplasm and translocates into the nuclei, where it interacts with its DNA-binding partner, known as T cell factor (TCF)/lymphocyte enhancer-binding factor 1 (LEF1), to stimulate the transcription of Wnt target genes [2, 16•, 51].

Wnt/β-catenin is an evolutionarily conserved cellular signaling system that plays an essential role in a diverse array of biologic processes, such as organogenesis, tissue homeostasis, and the pathogenesis of many human diseases [16•]. The contribution of Wnt/β-catenin signaling to fibrogenesis is becoming increasingly clear. Several studies suggest that the aberration of components of this pathway that fine-tunes the signaling is an essential driver of β-catenin-mediated fibrosis in fibrotic diseases. Activation of Wnt/β-catenin signaling has been reported in skin, kidney, liver, lung, and cardiac fibrosis [1••, 2].

With regard to cellular targets, several in vitro studies show that activation of Wnt/β-catenin signaling enhances proliferation, migration, and matrix production in human dermal fibroblasts, suggesting a key role for this signaling pathway in fibroproliferation and differentiation of fibroblasts into myofibroblasts [52, 53]. In addition to fibroblasts, epithelium is a notable target of Wnt/β-catenin signaling. In vivo, β-catenin is upregulated predominantly in renal tubular epithelium in fibrotic kidneys, and in vitro, activation of β-catenin in tubular epithelial cells induces EMT as well as the expression of several fibrosis-related genes, such as Snail1, plasminogen activator inhibitor 1 (PAI-1), MMP-7, and fibronectin [54, 55•, 56–58]. Likewise, activation of Wnt/β-catenin signaling is involved in mediating podocyte EMT, podocyte dysfunction, and glomerulosclerosis [59•]. Furthermore, many β-catenin target genes are key EMT regulatory transcription factors and mediators, including Snail, Twist, LEF1, and Jagged1 [16•]. Interestingly, recent studies demonstrate that tubular β-catenin can control the fate of interstitial fibroblasts via MMP-7-mediated epithelial–mesenchymal communication in renal fibrosis after obstructive injury [58]. Besides the aforementioned cell types, several studies indicate that a multipotent adipogenic progenitor that can be changed toward a fibrotic phenotype may be a critical target of Wnt/β-catenin signaling in fibrotic diseases [2]. By inhibiting adipogenic transcription factors CCAAT/enhancer-binding protein α and peroxisome proliferator-activated receptor γ [60], Wnt/β-catenin signaling antagonizes adipogenic gene expression and promotes dedifferentiation toward a phenotype of myofibroblast in both hepatic lipofibroblasts [61] and 3T3-L1 cells [2, 62].

Increasing evidence exists regarding crosstalk between Wnt/β-catenin and TGF-β. Wnt/β-catenin signaling can upregulate expression of TGF-β1 [52, 63], and TGF-β1 can activate β-catenin [56, 63, 64]. Smad3 knockout mice display less β-catenin activation, whereas a lack of β-catenin attenuates the ability of TGF-β to promote proliferation in fibroblasts [63]. β-Catenin and TGF-β can synergize to coregulate the same genes via Smad and TCF-binding sites within the promoter [65]. Although it is unclear whether Wnt/β-catenin signaling simply collaborates with the profibrotic factor TGF-β to promote matrix synthesis and assembly or it primarily governs cell fate decisions leading to abundant myofibroblasts through differentiation or transition from various other cell types during fibrogenesis, it is exciting to see that pharmacologic inhibition of Wnt/β-catenin signaling by different approaches is protective, resulting in amelioration of tissue fibrosis in models of fibrotic disease [2, 54, 58, 66•, 67, 68, 69•].

## Sonic Hedgehog Signaling

Hedgehog signaling is a highly conserved signaling pathway that orchestrates multiple aspects of embryogenesis, development, and tissue remodeling in a wide spectrum of systems [70, 71]. Hedgehog transmits its signaling by binding to the plasma membrane receptor, Patched (Ptc). In the absence of hedgehog, Ptc keeps the coreceptor Smoothened (Smo) in its inactive form and silences the Smo-dependent downstream intracellular signaling. When the extracellular microenvironment is enriched with soluble hedgehog, the interaction of hedgehog and Ptc leads to Ptc internalization and degradation, resulting in the derepression of Smo. Activated Smo moves from an intracellular vesicle to the cell membrane [72•], leading to an intracellular signaling cascade that ultimately drives the activation and nuclear translocation of glioblastoma (Gli) family zinc-finger transcription factors. The binding of Gli proteins to their cognate *cis*-acting elements regulates the expression of hedgehog target genes [73]. Gli1 and Gli2 mostly are responsible for providing prolonged cellular responses to hedgehog, whereas Gli3 primarily acts as a signaling repressor [74, 75]. Direct targets of hedgehog signaling contain several components in its own pathway, such as Ptc, Smo, Glis, and hedgehog-interacting protein 1, thereby providing both positive and negative feedback to ensure the delicate regulation of the pathway [72•, 76].

Among the three vertebrate hedgehogs, sonic hedgehog (Shh) is the best characterized and most widely studied. Shh signaling has been implicated in the regulation of injury repair and wound healing after tissue damage [72•, 77•]. Shh expression is upregulated in chronic fibrotic lung diseases, and the Shh signaling pathway may be involved in the remodelling of damaged pulmonary epithelium [78]. In mouse cholangiocytes, coculture with myofibroblastic hepatic stellate cells, a source of Shh, promotes EMT and cell migration, whereas addition of Shh-neutralizing antibodies to cocultures blocks these effects. Moreover, mice haploinsufficient for the Shh inhibitor Ptc exhibit increased Shh signaling activity, and their livers show enhanced fibrogenesis after bile duct injury and elevated expression of Gli2 and several mesenchymal markers [74, 79]. Thus, activation of Shh signaling promotes EMT and contributes to the evolution of biliary fibrosis during chronic cholestasis [74, 79]. In a mouse model of obstructive nephropathy, Shh is induced predominantly in renal tubular epithelium but targets interstitial fibroblasts. Either genetic Gli1 ablation or pharmacologic inhibition of Smo attenuates matrix gene expression and mitigates renal fibrotic lesions. This epithelial–mesenchymal communication, mediated by Shh/Gli1 signaling, probably plays a crucial role in the pathogenesis of renal fibrosis [72•].

Shh signaling elicits its action by regulating the transcription of its target genes [72•, 76]. The best-characterized direct target and downstream mediator of Shh is Gli1; another is Snail1, a key transcription factor for mediating EMT and fibroblast migration. Besides regulating its target genes, Shh might directly control the expression of a battery of fibrogenic genes, such as α-SMA, fibronectin, collagen I, and desmin, genes directly involved in myofibroblast activation and matrix production [72•]. It is worthwhile to note that through crosstalk with Wnt/β-catenin signaling and Notch signaling, Shh signaling can act in concert with other fibrogenic signaling pathways as well [76, 80], and the expression of Snail1 may be induced by both Wnt/β-catenin and Shh signaling [54, 59•, 72•]. Consequently, targeting Shh signaling might be a promising strategy for therapeutic intervention in a variety of fibrotic diseases.

## Notch Signaling

Notch proteins are single-pass transmembrane receptors with conserved expression among animal species during evolution. Their principal function is the regulation of many developmental processes, including proliferation, differentiation, and apoptosis. Mammals possess four different Notch receptors, referred to as Notch1–4. The Notch receptor consists of an extracellular domain, which is involved in ligand binding, and an intracellular domain that works in signal transduction. Notch ligands also are single-pass transmembrane proteins named Jagged (Jag1 and 2) and Delta (Dll1, 3, and 4) [81, 82]. Activation of this signaling pathway requires cell–cell contact. Interaction of ligands with the Notch receptors triggers a series of proteolytic cleavages, by a metalloprotease of the ADAM family (TACE; tumor necrosis factor-α-converting enzyme) and finally by the γ-secretase complex. The final cleavage leads to the release of Notch intracellular domain (NICD), which travels to the nucleus and binds to other transcriptional regulators (mainly of the CBF1/RBP-Jκ, SU(H), Lag1 family) to trigger the transcription of the target genes, classically belonging to the Hes and Hey family. This core signal transduction pathway is used in most Notch-dependent processes and is known as the canonical pathway [81, 83••, 84].

During the past few years, activation of Notch signaling has shown fibrogenic effects in a wide spectrum of diseases, including systemic sclerosis (SSc) [85•, 86], scleroderma, idiopathic pulmonary fibrosis (IPF) [87], kidney fibrosis [81, 88, 89], and cardiac fibrosis [83••].

Activated Notch1 and an elevated level of NICD are found in the lesional skin of SSc patients and in the skin and lungs of mouse models with SSc. Blocking the release of NICD with a γ-secretase inhibitor or treating SSc mice with Notch siRNA may reduce the collagen content in the skin and lungs [85•, 86, 90]. Activation of Notch signaling has major implications on fibroblast activation in SSc. Stimulation of SSc fibroblasts with a recombinant Jag-1-Fc chimera results in their differentiation into myofibroblasts overexpressing α-SMA and producing large amounts of ECM, whereas pharmacologic blockade of Notch signaling normalizes the proliferation rate of dermal fibroblasts extracted from lesional skin [85•, 90].

Activation of Notch signaling is associated with abnormal differentiation of respiratory epithelial cells in progressive IPF or secondary pulmonary fibrosis as observed in SSc. Hes-1, a Notch target gene, is upregulated in lung mucus cells from patients with chronic obstructive pulmonary disease, idiopathic pulmonary arterial hypertension, and IPF [87]. Activation of Notch signaling is critical for lung fibroblasts to differentiate into myofibroblasts. In bleomycin-induced lung fibrosis, Notch is indispensable in the upregulation of α-SMA induced by FIZZ1 (found in the inflammatory zone), a cysteine-rich secreted protein with fibrogenic properties, thus triggering the differentiation of fibroblasts into myofibroblasts [91]. Activation of Notch signaling induces expression of α-SMA, collagen I, and vimentin in alveolar epithelial cells in rats with lung fibrosis, which suggests that this signaling is involved in the EMT process, and Notch-mediated induction of Snail1 is required for TGF-β1-induced EMT in human alveolar epithelial cells [92, 93].

The participation of Notch in chronic kidney disease (CKD) has been studied in detail. Elevated levels of Notch ligands and receptors are detected in several glomerular diseases and tubulointerstitial fibrosis [81, 88, 89]. Either genetic deletion of a Notch transcriptional partner (CBF1), specifically in podocytes and tubular cells, or pharmacologic blockade of the Notch pathway with a γ-secretase inhibitor protects against glomerular injury and tubulointerstitial fibrosis in vivo [81]. Overexpression of Notch in renal tubular cells is necessary and sufficient for tubulointerstitial fibrosis development, and upregulation of Notch1 NICD (NICD1) increases ECM production in cultured tubular cells [94•]. NICD1 promotes tubulointerstitial fibrosis and glomerulosclerosis when overexpressed conditionally in tubular cells and podocytes in vivo, respectively [88, 95].

## Integrin-Linked Kinase Signaling

Abundant studies demonstrate that integrin signaling plays a critical role in the production and assembly of matrix proteins. Integrins are a family of heterodimeric transmembrane receptors containing α and β subunits. Integrins integrate signals between cells and their extracellular environment by connecting the cytoskeleton to the ECM. Because they have no enzymatic or actin-binding activity, integrins transmit their signals by activating their downstream effector kinases, focal adhesion kinase (FAK), and integrin-linked kinase (ILK) [5••, 16•, 96].

ILK can integrate and mediate several different intracellular signals associated with fibrogenesis, mainly because it is both a scaffolding protein and a serine/threonine protein kinase. Ectopic expression of ILK directly phosphorylates GSK-3β, then the inactivation of GSK-3β leads to stabilization of Snail1 and β-catenin. Snail1 regulates EMT and fibroblast motility, whereas β-catenin directly governs the transcription of fibronectin and PAI-1. On the other hand, as a scaffolding protein, ILK assembles a multicomponent protein complex that contains ILK, PINCH, and parvin [5••, 14, 97]. These characteristics of ILK enable it to bridge the integrins, actin cytoskeleton, and other growth factor signal cascades [5••]. It is worth noting that the integrity and activity of this complex are necessary for ECM production, because either inhibition of ILK activity or destruction of this complex attenuates ECM expression, deposition, and extracellular assembly [14, 96, 98, 99]. Several critical fibrogenic signals induce ECM production by regulating this complex. For instance, TGF-β1 promotes induction of β1-integrin, ILK, and PINCH1, as well as their assembly. CTGF binds to both integrins and LDL receptor-related protein 1 (LRP1), resulting in the activation of ILK and production of matrix components. PDGF and FGF2 signaling are potentially linked to this complex via PINCH1. Tissue-type plasminogen activator promotes collagen I expression by triggering LRP1-mediated β1-integrin recruitment and ILK activation. Angiotensin II induces TGF-β1 expression, activates Smad signaling, and induces β1-integrin and ILK expression. Therefore, this molecular complex functions as a platform that integrates various fibrogenic signals and controls ECM production [5••].

## Conclusion

Given the diversity of known fibrogenic mediators, the intracellular signal cascades involved might be immensely complex, with almost immeasurable crosstalk and feedback. In essence, the activation of major fibrogenic signaling mentioned earlier is characteristic of various fibrotic diseases. During the past decade, our understanding of the profibrotic mechanisms of these signaling pathways has evolved and improved considerably. It seems intriguing and meaningful to identify some of the converging molecular machineries that integrate various signal inputs and control the transcriptional program for myofibroblast activation and matrix production. Because several strategies to target key profibrotic mediators and signaling pathways to block the process of fibrosis are effective in different animal models and clinical trials [1••, 5••, 100••, 101••], therapeutic strategies based on these observations will bring new hope to clinicians and patients in the future.
